# Optimization of pediatric CT scans in a developing country

**DOI:** 10.1186/s12887-021-02498-2

**Published:** 2021-01-20

**Authors:** Fotso Eddy Kamdem, Samba Odette Ngano, Clemence Alla Takam, Alain Jervé Fotue, Serge Abogo, Cornellius Lukong Fai

**Affiliations:** 1 Unité de Recherche de la Matière Condensée, d’Electronique et de Traitement du Signal, Department of Physics, Faculty of Science, University of Dschang, Dschang, Cameroon; 2 Radiography Department, Yaoundé General Hospital, Yaoundé, Cameroon; 3 Department of Radiology, National Social Insurance Fund Hospital, Yaoundé, Cameroon

**Keywords:** Pediatric CT scanner, Optimization of protocols, Dose reduction

## Abstract

**Background:**

The purpose of this study was to assess scan parameters and to propose strategies to optimize the examinations of children (from 0 to 15 years old) on adult scanners in developing countries.

**Methods:**

A study was done in 2015 and 2018 on 312 pediatric patients to verify improved practices. The study of 2015 ended with proposed strategies. Dose and scan parameters were available for prospective dose analysis. These strategies were implemented in a study of 2018.

**Results:**

Amount the CT examinations study in this paper, the common was head trauma (90 %). For every pediatric CT scan in 2015, a kV of 120 was used in the various hospitals. The mAs ranged from 57.75 to 283.33, slice thicknesses from 1.25 to 2.5 mm and pitch from 0.525 to 1.375 mm. In the study of 2018, implementing the strategy defined in the methodology and proposed in 2015: CTDI_Vol_ decreased by 21.27 % for children < 1 year, 31.97 % for children 1–4 years, 17 % for children 5–9 years. DLP also decreased by 25.14 %, 36.29 % and 19.85 % for children < 1 year, 1–4 years and 5–9 years respectively. Children were exposed to ionizing radiation on machines designed for adults, but now the doses received by children are reduced.

**Conclusions:**

The reduction of doses during the pediatric CT examination is possible with the introduction of new optimization protocols or the acquisition of a new machine with a pediatric protocol.

## Main points

- In some developing countries such as this country, pediatric CT scanners are made on adult CT scanners that do not have pediatric protocol available.
- Displaying an example of a pediatric protocol (procedures, doses, kV and mA recommended for example) in the CT scanner control room allows medical imaging technicians to optimize pediatric exams on adult-designed CT scanners.
- Propose strategies to optimize the examinations of children (from 0 to 15 years old) on adult CT scanners in developing countries.

## Background

CT is a medical imaging modality with superior image quality and diagnostic capability compared to conventional radiology [1, 2]. It is the most used imaging modality in this country for imaging brain trauma. The CT scanner is a technic, which better diagnoses certain head pathologies [1, 2]. Pediatric radiology requires adequate equipment, specific precautions and specialized knowledge of ionizing radiation. This is not an easy task for developing countries, especially regarding equipment and precautions. This difficulty is due to multiple problems (poverty) encountered in most African countries. In these countries, older generation CT scanners for adults are the most used.

Exposure to ionizing radiation is risky: This is why it is important not to expose patient to unnecessary ionizing radiation. This notion of patient exposure concerns the scientific community. Therefore, several attempts have been made to develop solutions to restrict or eliminate unnecessary radiation that a child receives during a CT scan. The dose reduction will therefore initially be done via the evolution of the technologies developed by the manufacturers (iterative reconstructions, dose modulation techniques, etc.) as well as adapting acquisition protocols in order to obtain a diagnostic quality image for the lowest dose level possible [1]. The optimization of protocols in pediatric imaging is essential, even capital, because each CT has specific technical specificities. The protocols are not directly transferable from one CT to another even if the approach of optimization can be the same [1].

The protection of persons exposed to ionizing radiation (IR) for medical purposes is well regulated in developed countries (France) by Decree No. 2003 − 270 of 24 March 2003, issued from the Directive 97 − 43 of Euratom Council of 30th June 1997 on the protection of the health of individuals against the dangers of IR on the grounds of medical exposure [2]. In this country, we do not yet have a Decree, a legal or regulatory text for the protection of persons exposed to IR. This explains the lack of a specific national dose reference levels. The need to minimize the doses received during the examinations is essential in diagnostic radiology and even more so in pediatric CT. As the child is very small as compared to adults, the dose he receives from the adult protocol is inappropriate for his morphology. In this context, the optimization of the irradiation dose and pediatric protocols should not be neglected on those machines. The general objective of this study was to assess the parameters of radiological protocols and to propose strategies to optimize the examinations of children on adult scanners in developing countries. It also included a comparative study of the radiological protocol parameters studied in 2015 and 2018 in order to observe the improvement of pediatric CT scan practice in hospitals in the country.

## Materials and method 

### Patients characteristics

After conducting a retrospective study for a period of 6 months (February to July 2015) 4 years ago with 212 head trauma cases, another prospective and observational 2-month study was conducted including other 100 cases (from September to October 2018) on pediatric patients aged 0 to 15 years in a public (H1), parastatal (H2) and private (H3) hospital in this developing country. The age groups are : <1 year, 1–4 years, 5–9 years and 10–14 years. This study was approved by the research ethics board of the included three hospitals with the protocol number of Y15/01. Patient consent was obtained. Parents’ verbal consent has been given for all minors. Patients without detailed radiation dose reports were excluded from the study.

### CT scan protocols

Parameters such as CTDI_Vol_, DLP, tube current-time product (mAs), High tube voltage (kV), Age, Gender, slice thickness (T), the mode and the pitch of each child for each type of scan were recorded during the skull exam. Although the image quality has not been assessed in this study, only diagnosis quality scanner was included. We obtained the parameters relevant to radiation dose from the scan protocol generated by the three different CT systems (2 General Electric (Brightspeed 8 slice and Lightspeed 16 slice), and one Hitachi Eclos 16 slice) from the three centers after each cranial CT.

#### Before optimization

In 2015, hospitals used the helical mode and the same kV for all age groups. The tube current-time product and the slice thickness (T) used, were according to the default values offered by the CT scanners. The reconstruction was not used to reduce the absorbed doses of examinations. Radiologists used the automatic reconstruction parameters of the CT scanner and the same filters were used for the same exams (trauma). The CT scanners studied do not have pediatric protocol. Many imaging technicians were not sensitized on the concepts of optimizing dose protocols, quality control and the notion of radioprotection especially on children.

#### Proposed strategies

The major findings of our study were as follows; Realize Continuing Professional Development (CPD) for imaging technicians on the concepts of optimizing dose protocols, and the notion of radioprotection especially on children; Realize quality control on CT scanners; Use the reconstruction to reduce the absorbed doses of examinations; Not always use automatic reconstruction parameters of the CT scanner and the same filters for the same exams; Introduce pediatric protocol in CT scanners; Reduce the acquisition parameters according to the patient’s age; Display DRL in the CT scanner control room.

#### Methodology (strategies) between 2015 and 2018

Hospitals still used the helical mode but the high tube voltage change. The tube current-time product, and slice thickness also varied positively to dose reduction. Hospital allow an engineer to collaborate for the change of the protocols (Their CTDI_vol_ are now below 51.94 mGy) in the scanner at H_3_. The first radiology quality control seminar was organized in September 2017, where each hospital in this study sent at least one medical imaging technician and radiologist. The concepts of optimizing dose protocols, quality control and the notion of radioprotection especially on children, were included in the seminar training.

### Statistical analysis

Statistical analysis was performed using Microsoft Excel software. Dose and scan parameters were expressed as mean, range and third quartile (CTDI_Vo_l and DLP).

## Results

Three hundred twelve patients participated in this study, for 460 acquisitions. The most studied CT scan protocol in 2015 and 2018 was trauma protocol. Table 1 presents the number of acquisitions according to the different age groups in these 3 hospitals.

**Table 1:** Total number of skull examinations and age groups in 2015 and 2018

Years	Examination	Number of acquisitions			
2015	Age	< 1 year	1–4	5–9	10–14
	Skull (trauma)	63	60	55	95
2018	Age	< 1 year	1–4	5–9	10–14
	Skull(trauma)	45	40	57	45

In 2015, the helical mode and the same high tube voltage of 120 kV were used for all age groups. Tube current-time product (mAs) and slice thickness (T) ranged from 57.75 to 283.33 mAs and 1.25 to 2.5 mm respectively (Table 2). The principles of radioprotection were disregarded by medical imaging technicians because after entering the patient’s characteristics, they did not change the CT scanner settings before doing the examination. Child or adult, they used the same scan parameters. Effort was not made to use reconstruction parameters for the dose optimization. Radiologists used the automatic reconstruction (software) inherent to the CT scanner. The same filters were used for the same exams (trauma). That is why all these hospitals used small slice thickness (1.25–2.5 mm).

**Table 2:** Comparison of Skull (Trauma) radiological protocol parameters, CTID_V_ (mGy) and DLP (mGy.cm) for one acquisition in the various hospitals in 2015

Skull exami-nation/ age	Hospi-tal	kV mean (min-max)	mAs mean (min-max)	T(mm) mean (min-max)	Pitch mean (min-max)	CTDI_V_ mean (min-max)	DLP mean (min-max)	Mode
< 1	**H**_**1**_	120 (120–120)	175 (175–175)	2.5 (2,5 − 2 ,5)	0.94 (0,94 − 0,94)	37.1 (37.1–37.1)	1061.06 (368.77–1298,5)	Helical
	**H**_**2**_	120 (120–120)	280 (280–280)	1.75 (1.25–2.5)	0.625 (0.62–0.62)	46.99 (5.25–51.94)	963.70 (211.4-1193.8)	Helical
	**H**_**3**_	120 (120–120)	150 (150–150)	1.25 (1.25–1.25)	0.562 (0.56–0.56)	43.44 (43.44–43.44)	580.93 (580.9-580.93)	Helical
1–4	**H**_**1**_	120 (120–120)	175 (175–175)	2.5 (2,5 − 2 ,5)	0.94 (0,94 − 0,94)	37.1 (37.1–37.1)	1209.46 (1209.46-1209.46)	Helical
	**H**_**2**_	120 (120–120)	280 (280–280)	2.25 (2.25–2.25)	0.625 (0.62–0.62)	49.46 (37.45–51.94)	941.51 (696.27-1206.87)	Helical
	**H**_**3**_	*-*	-	*-*	-	*-*	-	-
5–9	**H**_**1**_	120 (120–120)	175 (175–175)	2.5 (2,5 − 2 ,5)	0.94 (0,94 − 0,94)	37.1 (37.1–37.1)	1143.79 (1143.79-1143.79)	Helical
	**H**_**2**_	120 (120–120)	224 (224–224)	1.65 (1.25–2.5)	0.625 (0.62–0.62)	42.96 (37.1-51.94)	1138.05 (888.84-1518.52)	Helical
	**H**_**3**_	120 (120–120)	280 (280–280)	1.25 (1.25–1.25)	1.375 (1.37–1.37)	46.44 (46.44–46.44)	822.13 (822.13-822.13)	Helical
10–14	**H**_**1**_	*-*	-	*-*	-	*-*	-	-
	**H**_**2**_	120 (120–120)	262,53 (149–280)	2.12 (1.25–2.5)	0.625 (0.62–0.62)	39,06 (22.39–54.94)	859.65 (643.97-1280.63)	Helical
	**H**_**3**_	*-*	-	*-*	-	*-*	-	-

In 2018, after the optimization techniques recommended in 2015 in these hospitals, the kV are now modified and vary from 100 to 120 kV, depending on the ages and the thickness of the children. The mAs are modified before the examination (100 to 250 mAs), thickness from 2 to 2.5 mm and 0.625 is generally use for the pitch. The use of reconstruction parameters for dose optimization allows to use 2-2.5 mm slice thickness with new CT technology for the first acquisition. The activation of the pediatric protocol option in the CT scan software is now effective in hospital H3 and is one of the key factors in optimizing the practice of CT scan.

Table 3 compares the 3rd quartiles of skull CTDI_Vol_ and skull DLP of this study in 2015 and 2018 for an acquisition in some European countries (Switzerland [3], Germany [4], France [5], and Ukraine [6]. The CTDI_Vol_ values of children < 1 year of this study (43.44 mGy) are superior to the comparative literature (20 mGy for Switzerland). The DLP (mGy.cm) of this country (1200.04 in 2015 and 961.72 in 2018) are higher than all those of literatures (900 for Shrimpton ) for children aged from 5 to 9 years. CTDI_Vol_ of pediatric skull aged 1–4 years in 2018 (35.33 mGy) are below the one of some comparative countries (45 mGy for Ukraine, 40 mGy for Germany and France). Table 3 shows that the 3rd quartile of the doses of this study are very high compare to the Diagnostic Reference Levels (DRL) of France of 30th May 2019[7] and those of Shrimpton and al [6].

**Table 3:** Comparison of the 3^rd^ quartiles of the CTDI_V_ (mGy) and DLP (mGy.cm) in 2015 and 2018 of this study, with international studies for the skull (Trauma) for one acquisition

Dose values	CTDI_V_			DLP		
**Examination/** **Ages**	Skull /<1	Skull /1–4	Skull /5–9	Skull /<1	Skull /1–4	Skull /5–9
**This study (2015)**	43.44	51.94	51.94	895.21	1141.63	1200.04
**This study (2018)**	34.2	35.33	43.11	670.1	727.3	961.72
**Switzer-land 2008** [3]	20	30	40	-	-	-
**Germany 2007** [4]	33	40	50	-	-	-
**France 2009** [5]	30	40	50	-	-	-
**Ukraine 2005**[6]	30	45	50	-	-	-
**DRL France(2019)**[7]	20	22	26	320	360	470
**Shrimpton and al** [6]	-	-	-	420	600	900

Compared to 2015, radiation dose results for 2018 are lower (Table 4). CTDI_Vol_ decreased by 21.27 % for children < 1 year, 31.97 % for children 1–4 years, 17 % for children 5–9 years, and DLP also decreased by 25.14 %, 36.29 % and 19.85 % for children < 1 year, 1–4 years and 5–9 years respectively for skull (trauma) examinations.

**Table 4:** Comparison of the 3^rd^ quartiles of the CTDI_vol_(mGy) of that study for the skull (Trauma) in 2015 and 2018 for one acquisition

Exam/ages	Dose value/ Age		Hospitals		
			H_**1**_	H_**2**_	H_**3**_
**Skull<1**	CTDI_vol_	2015	37.1	51.94	48.99
		2018	-	-	34.2
**Skull /1-4**	CTDI_vol_	2015	37.1	51.94	48.98
		2018	-	35.3	34.2
**Skull /5-9**	CTDI_vol_	2015	37.1	51.94	51.94
		2018	-	45.04	37.32

## Discussion

The major findings of our study were as follows; the doses decreased from 2015 to 2018 up to 36.29 %. The CTDI_Vol_ of this study in 2018 are lower for skull examinations of 1 to 4 and 5 to 9 years (Table 3) than those of Germany, France and Ukraine. For children < 1 year, the values of this study are higher. Furthermore, cranial CT dose and dose parameters were significantly variable between centers due to the incompatible CT protocols which would be standardized (Table 2). This shows that improvements are still possible.

Table 4 shows that the CTDI_Vol_ values in this study have decreased. The introduction of the pediatric protocol in the scanner at H_3_ was done by an engineer in collaboration with the team of this study. Additionally, a protocol to follow is posted by the team in the control room scanner. H_2_ continues to use the proposed protocol and recommendations on their old CT scanner. In 2018, after our second study, H_3_ bought a new machine with the pediatric protocol of the functional head. H_1_ is closed.

The results above show that we still need to improve pediatric exams on this type of CT scanner. This is the reason why technicians and radiologists need to be trained in pediatric protocols harmonization and optimization. In this way, it will be possible to reduce kV and mAs, use reconstruction technique and limit the number of acquisitions. In 2015 majority of these technicians had not received training in radiation protection. As the pediatric protocol was not functional in their machine, they validated most of the default values offered by the machine for pediatric examinations when they were present. The dose ratios of adults and children were therefore approximately identical. Some even did not know that kV could be modified as in conventional radiology although they know how to introduce the parameters of the patient, to start the acquisition, to reduce the lengths of the scout view, to stop the examination. However, this is not enough to optimize a protocol in a CT and can lead to deliver large amounts of doses to children.

The results of the study in 2015 (comparison with international literature) allowed us to propose strategies to reduce the doses absorbed by children on these CT scanners. In 2015, the hospitals all used the helical mode and the same 120 kV for all age groups as for adult patients. However, the reasoning in pediatric CT should be different from that used in adults where, to reach the useful dose levels, kV between 110 and 140 are used. In 2018, from the study by Heliot C., Mestdagh P., Opsomer H., Chaffiotte C., we showed the CT scanner operators that reducing the kV from 120 to 80 reduces the dose delivered by a factor 2.2 [8]. The choice of a relatively low value should be preferred (one can use 80 kV for newborns) because it helps to reduce the dose [9]. As for the International Commission on Radiological Protection [10] (ICRP), for children weighing between 5 and 50 kg, it is possible to use 100 kV in the routine. Compared to adult protocols, the mAs must be reduced according to the morphotype. This is possible without significantly altering the intrinsic quality of the image [11]. A reduction of milli-Amperage by two thus makes it possible to reduce the dose by two but increases the noise of the image by a factor of 1.4 [12]. This is possible without significantly altering the intrinsic quality of the image [11].

Table 2 shows that there are disparities in the values of​​ slice thickness (T) and pitch in our hospitals. Constant noise, the acquisition in this section is at the origin of an increase in irradiation dose [13]. In case of excessive reduction of the milli-Amperage, the acquisition in fine slice generates a significant noise increase. It is true that the small size of children requires thinner slice thicknesses than adults but a reduction in the slice thickness leads to an increase in the irradiation dose. A high pitch, of the order of 1.5, will be preferred to reduce acquisition time and movement artifacts (for example, when exploring a poly-traumatized patient). The pitch must, however, remain below 2 in order to keep the quality of multi-planar reformations [14] optimal and to avoid the appearance of propeller artifacts [15]. The medical imaging technicians use CT scanners working at “constant voltage” and should in principle be careful when changing the pitch as this could automatically change the mA. It is then necessary to readjust the mAs secondarily to obtain the CTDI_Vol_ optimized for each type of examination because the irradiation dose is directly proportional to the tube current-time [11]. In 2015, radiologists used the automatic reconstruction (reconstruction software) parameters of the CT scanner. The same filters were used for the same exams (trauma). Reconstruction was not used to reduce the dose but to have a good image, by reducing the noise. But as in 2018, with new CT scanner (H_3_), it is used to reduce the dose.

Today, it is possible to use large slice thickness (5 mm) that can be reduced during the reconstruction to 1.25, or 2.5 mm. The reconstruction of the images is an important technical factor that is modifiable after the acquisition and which influences the quality of the images and thus indirectly the dose. For lack of time, the medical imaging technicians use the helical mode in 2015 and in 2018. The mode of acquisition is one of the technical factors that influences the dose and is accessible at the time of acquisition. The helical mode has significantly reduced the acquisition time. For the same acquisition length, the area exposed to ionizing radiation is larger in helical mode than in sequential mode [12].

Parameters such as: mAs, slice thickness and pitch in protocols used were different across hospitals. This explains the differences in values in those hospitals. A common procedure for the use of CTs for children must be set up in this country. The existing protocols in CT scanners, for acquisition and for reconstruction, do not have any modification whether adult or children, thin or obese patient. Some protocol parameters can be modified directly by the user before acquisition (acquisition mode, kV, mA, detector number and pitch) and others after acquisition (slice thickness, the inter-slice interval, the reconstruction algorithm and the reconstruction filter) to optimize the dose and the image quality, either at the time of acquisition, or at posteriori [12]. The first possibility of protocol modification is mostly used in 2018 in these hospitals. The presence of an experienced radiologist at the console would be necessary to know how to justify each sequence and stop the exploration when the information is obtained. The indication in the patient’s request for examination should be given increased attention prior to acquisition. This is the base line for scan length reduction and number of acquisition reduction. It is true that we do not have enough radiologists in our hospitals, but those who are in positions must make the maximum effort to be present during the CT examinations, or to train their medical imaging technicians on how to stop an examination when the purpose of the prescriber’s request is met.

Many medical imaging technicians in 2015 were not sensitized on the concepts of optimizing dose protocols and the notion of radioprotection especially on children. At the first radiology quality control seminar in September 2017, each hospital in this study sent at least one medical imaging technician and radiologist. Medical imaging technicians needed to be educated to improve their pediatric examination on older generation CT scanners. After invitation to the first radiology quality control seminar in September 2017 by an author of this article, they were educated on the concept of radiation protection and the quantities of doses delivered by their CT scanner. This seminar was realized with a large contribution of the International Atomic Energy Agency (IAEA) through his expert in radiology (Dr. Marco BRAMBILLA), that we will like to thank. The knowledge and skills of medical imaging technicians and radiologists must be updated through seminars, continuous and regular training in the radiological protection field.

Our study has several limitations. First, we were not able to find some of the CT acquisition parameters in the dose report of CT examinations, which limited to evaluate the effects of each parameter on CT doses. The second limitation was the number of CT with mechanical failures. The third limitation was the unavailability of different parameters such as tube filtration and detector configuration, all of which vary across vendors. The fourth limitation was the fact that image quality has not been assessed. Although we emphasized the importance of modifying CT acquisition parameters such as decreasing tube voltage or increasing tube rotation time to reduce radiation dose, we did not evaluate the effect of modification of these parameters on image quality.

## Conclusion

This study presents scan and dose parameters of CT in a developing country for skull (trauma). In 2015 majority of medical imaging technicians had not received training in radiation protection and quality dose control. Scan parameters were not modified during the examinations. After the implementation of our recommendations in these hospitals, the scan and dose parameters are now modified depending on the age and thickness of the children. In 2018, dose parameters are decreased. Some hospitals have finally purchased CT with both protocols (adult and pediatric) functional. The analysis of these data proves that the protocols used on children at CT in this survey have been improved from a radiation safety point of view. However, improvements are possible by using image reconstruction, reducing scan lengths by adjusting the scan length to the exam’s indication, choosing the right acquisition mode, activating pediatric protocol in CT scanners in the country.

## Data Availability

Data is available upon request.

## Abbreviations

CPD: Continue Program Development
kV: High tube voltage
mAs: Tube current-time product
H: Hospital
CTDI_Vol_: Volume CT Dose Index
DLP: Dose-Length Product
CT: Computed Tomography
IR: Ionizing radiation
T: Sclice thickness
ICRP: International Commission on Radiological Protection
IAEA: International Atomic Energy Agency
