# Correction to: Optimization of pediatric CT scans in a developing country

**DOI:** 10.1186/s12887-021-02555-w

**Published:** 2021-02-24

**Authors:** Eddy Fotso Kamdem, Odette Ngano Samba, Clemence Alla Takam, Alain Jervé Fotue, Serge Abogo, Cornellius Lukong Fai

**Affiliations:** 1 Department of Physics, Faculty of Science, Unité de Recherche de la Matière Condensée, d’Electronique et de Traitement du Signal, University of Dschang, Dschang, Cameroon; 2 Radiography Department, Yaoundé General Hospital, Yaoundé, Cameroon; 3 Department of Radiology, National Social Insurance Fund Hospital, Yaoundé, Cameroon

**Correction to: BMC Pediatr 21, 44 (2021)**

**https://doi.org/10.1186/s12887-021-02498-2**

Following the publication of the original article [1], the Conflict of interest statement tagging does not follow tagging guidelines to include an attribute to make it machine-readable.

Tagging error. The <DataAvailability> element includes the Data Availability, Ethics, Consent for publication, and Competing Interests statements.

An <Ethics> element is missing; it should hold the Ethics, Consent for publication, and Competing interests statements.

The original article has been corrected.
